# Predictors of health workers’ knowledge about artesunate-based severe malaria treatment recommendations in government and faith-based hospitals in Kenya

**DOI:** 10.1186/s12936-020-03341-2

**Published:** 2020-07-23

**Authors:** Beatrice Machini, Dejan Zurovac, Beatrice Amboko, Lucas Malla, Robert W. Snow, Hillary Kipruto, Thomas N. O. Achia

**Affiliations:** 1 University of Nairobi, Institute of Tropical and Infectious Diseases, Nairobi, Kenya; 2 Division of National Malaria Programme, Ministry of Health, Nairobi, Kenya; 3 KEMRI-Welcome Trust Research Programme, Nairobi, Kenya; 4 Centre for Tropical Medicine and Global Health, University of Oxford, Oxford, UK; 5 World Health Organization, Nairobi, Kenya; 6 School of Mathematics and Computer Science, University of Kwa Zulu Natal, Durban, South Africa

**Keywords:** Artesunate, Knowledge, Severe malaria, Predictors

## Abstract

**Background:**

Health workers’ knowledge deficiencies about artesunate-based severe malaria treatment recommendations have been reported. However, predictors of the treatment knowledge have not been examined. In this paper, predictors of artesunate-based treatment knowledge among inpatient health workers in two hospital sectors in Kenya are reported.

**Methods:**

Secondary analysis of 367 and 330 inpatient health workers randomly selected and interviewed at 47 government hospitals in 2016 and 43 faith-based hospitals in 2017 respectively, was undertaken. Multilevel ordinal and binary logistic regressions examining the effects of 11 factors on five knowledge outcomes in government and faith-based hospital sectors were performed.

**Results:**

Among respective government and faith-based health workers, about a third of health workers had high knowledge of artesunate treatment policies (30.8% vs 32.9%), a third knew all dosing intervals (33.5% vs 33.3%), about half knew preparation solutions (49.9% vs 55.8%), half to two-thirds knew artesunate dose for both weight categories (50.8% vs 66.7%) and over three-quarters knew the preferred route of administration (78.7% vs 82.4%). Eight predictors were significantly associated with at least one of the examined knowledge outcomes. In the government sector, display of artesunate administration posters, paediatric ward allocation and repeated surveys were significantly associated with more than one of the knowledge outcomes. In the faith-based hospitals, availability of artesunate at hospitals and health worker pre-service training were associated with multiple outcomes. Exposure to in-service malaria case-management training and access to malaria guidelines were only associated with higher knowledge about artesunate treatment policy.

**Conclusion:**

Programmatic interventions ensuring display of artesunate administration posters in the wards, targeting of health workers managing adult patients in the medical wards, and repeated knowledge assessments are likely to be beneficial for improving the knowledge of government health workers about artesunate-based severe malaria treatment recommendations. The availability of artesunate and focus on improvements of nurses’ knowledge should be prioritized at the faith-based hospitals.

## Background

Despite a falling prevalence of *Plasmodium falciparum* infection [1, 2] severe malaria is a common cause of admission in Kenyan hospitals [3]. Alongside the World Health Organization (WHO) recommendation in 2012 [4], Kenya was among the first African countries to adopt the artesunate treatment policy for severe malaria [5]. The Division of National Malaria Programme revised national malaria guidelines to reflect the new policy, procured and facilitated distribution of injectable artesunate to health facilities, developed training curriculums and job aids around new case-management standards, and implemented in-service training programs to facilitate readiness of clinicians and nurses to deliver new treatment standards [6, 7]. Of estimated 400 public hospitals in the country, about three-quarters are government-owned and the remaining are faith-based organization (FBO) hospitals [8]. National artesunate implementation equally targeted government and FBO health workers, but subsidized artesunate was available only to the government hospitals procuring medicines through the Kenya Medical Supply Agency.

According to the national guidelines [5], artesunate is the recommended treatment for all patient populations with severe malaria including pregnant women across all trimesters of the pregnancy. The recommended dose is 3 mg/kg for children below 20 kg and 2.4 mg/kg for patients above 20 kg. For the inpatient management, the preferred route of artesunate administration is intravenous with 12 hourly dosing intervals between the first three doses. After the minimum of three parenteral doses artesunate should be continued at 24 hourly dosing intervals until the patient is able to take oral anti-malarial therapy.

Health workers’ knowledge about new treatment policy and recommended use of the new medicines is one of the basic pre-requisites determining the readiness of the health system to implement any drug policy [9–11]. Several studies have suggested major knowledge deficiencies about artesunate-based treatment recommendations [12, 13], but no study has examined predictors of the health workers’ knowledge. This study examined the predictors of the inpatient health workers’ knowledge about artesunate-based severe malaria treatment recommendations in government and FBO hospitals in Kenya.

## Methods

### Data sources

The secondary analysis of two rounds of cross-sectional, inpatient malaria case-management surveys undertaken at government hospitals in 2016 and two rounds of the cross-sectional surveys undertaken in 2017 at the FBO hospitals was performed. All of 47 Kenyan Counties with a government referral hospital and 43 of 47 Counties with a major FBO hospital (four Counties did not have a FBO hospital) were included. Of 90 surveyed hospitals across both sectors, 26 are in high malaria risk areas around the Lake Victoria and along the Indian Ocean coast while the remaining 64 hospitals are within the low risk areas [2, 14]. All hospital surveys applied the same methodologies and the details have been previously published [12]. Of relevance for predictors of the health workers knowledge, interviews with randomly sampled clinician and nurse on duty during the day shift in each of the admission wards of interest (paediatric and combined medical wards) were undertaken following fishbowl (out of hat) sampling technique. During the interviews data on health worker’s demographics, exposure to in-service trainings, guidelines, supportive supervision, and their knowledge about management of severe malaria were collected. The knowledge component was assessed using self-administered, multiple choice questions. The interviews were undertaken by experienced external hospital nurses trained in the week prior to data collection and using pre-tested data collection form upon obtaining written informed consent from the participant. Regarding the hospital level factors, they included the physical assessments of the non-expired artesunate at the pharmacy and the presence of displayed artesunate administration posters in the wards. Upon the completion of the interviews and knowledge assessments, all participating health workers were informed about the correct responses, given national malaria case-management guidelines, and if absent, distributed artesunate administration posters to be displayed on the walls.

### Knowledge outcomes and definitions

Five knowledge outcomes reflecting severe malaria treatment recommendations based on artesunate use were selected. The outcomes reflected the correctness of health workers’ knowledge about (1) severe malaria treatment policy, (2) artesunate dose, (3) dosing intervals, (4) artesunate preparation, and (5) preferred route of artesunate administration. All outcomes were categorized on three-point scale into high, medium and low knowledge levels with an exception of the preferred route of administration which had two levels of the categorization. The definitions of the knowledge categories for each outcome assessed are provided in Table 1.

**Table 1:** Categories of the knowledge outcomes and study definitions

Knowledge outcomes	National recommendations	Knowledge categories	Category definitions
Treatment policy for severe malaria	Artesunate for the following 3 severe malaria populations Children and non-pregnant adults; Pregnant women in 1st trimester Pregnant women in 2nd & 3rd trimester	High	Artesunate response for all 3 severe malaria populations
		Medium	Artesunate response for 2 severe malaria populations
		Low	Artesunate response for one or none of the populations
Artesunate dose	2 Weight categories 3 mg/kg for child < 20 kg, 2.4 mg/kg for patient > 20 kg	High	Correct response for 2 weight categories
		Medium	Correct response for one weight category
		Low	No correct response for any of the weight categories
Artesunate dosing interval	3 dosing intervals 12 h between 1st and 2nd dose 12 h between 2nd and 3rd dose 24 h between 3rd and 4th dose	High	Correct response for all 3 dosing intervals
		Medium	Correct response for 2 dosing intervals
		Low	Correct response for one or none of the dosing intervals
Artesunate preparation	Solutions for 2 artesunate preparation steps Bicarbonate for reconstitution Saline or 5% dextrose for dilution	High	Correct response for 2 preparation steps
		Medium	Correct response for one preparation step
		Low	No correct response for any of the preparation steps
Preferred route of artesunate administration	Intravenous slow bolus	High	IV slow bolus response
		Low	Any other response

### Statistical analysis

Descriptive analyses, disaggregated by predictor variables, were undertaken using frequencies and percentages for each of the five outcomes. To examine health worker and health facility factors associated with health workers’ knowledge about severe malaria treatment, multilevel ordinal and binary logistic regression models were used for four ordinal and one binary outcomes respectively (Table 1). The examined health worker level factors included gender, age, pre-service training, ward allocation, and exposure to malaria guidelines, in-service malaria case-management training and supportive supervision. While the hospital-level factors included the availability of artesunate during the survey, display of artesunate administration posters and endemicity. Multilevel modelling approach was adopted to account for clustering of health workers within the hospitals. Candidate predictor variables were selected using unadjusted regression models for each outcome, with multicollinearity tested between those that had *P*–values < 0.15. To obtain adjusted estimates, the selected variables were included in multivariable regression models. Survey round was adjusted for in all regression models. The assumption of proportional odds in the final multivariable ordinal regression models was examined using Brant test [15]. As the surveys in government and FBO hospitals were conducted in different years all analyses were stratified by hospital ownership. The results from the multivariable models are presented as adjusted odds ratio (aOR) with *P*-values and 95% confidence intervals (CI). All analyses were conducted using Stata version 14 (StataCorp, College Station, TX, USA). Hypothesis testing and CI estimations were done with significance level of 0.05.

## Results

### Characteristics of study health workers

Table 2 presents characteristics of 367 and 330 health workers respectively interviewed at the government and FBO hospitals. In both sectors, most health workers were female, younger than 35 years, having less than 5 years of inpatient experience and working in low malaria risk areas. Nurses and clinicians as well as paediatric and medical ward health workers were similarly represented within and between hospital sectors. Compared to FBO sector, government health workers were however more commonly female (61.9% vs 51.2%), older than 35 years (37.6% vs 17.3%) and with more than 5 years of experience (43.9% vs 24.5%). Regarding the exposure to the relevant interventions, over three-quarters of health workers in both sectors worked at hospitals with artesunate in stock however, only about a third had access to malaria guidelines and less than a quarter received in-service malaria case-management training and supportive supervision in past 3 months (Table 2). While only minor differences were observed between the sectors with respect to the training and supervision, government health workers however less commonly had access to malaria guidelines (32.2% vs 39.8%) but more frequently worked in wards with displayed artesunate administration poster (61.0% vs 47.0%).

**Table 2:** Characteristics of study health workers

Health worker characteristics	Government hospitals		Faith based hospitals	
	N = 367		N = 330	
	n	%	n	%
Gender				
Male	140	38.1	161	48.8
Female	227	61.9	169	51.2
Age (years)^a^				
35–70	138	37.6	57	17.4
21–35	229	62.4	271	82.6
Pre-service training				
Clinician	175	47.7	156	47.3
Nurse	192	52.3	174	52.7
Inpatient experience (years)^b^				
< 5	203	55.8	249	75.5
> 5	161	44.2	81	24.5
Ward allocation				
Paediatric	185	50.4	168	50.9
Medical	182	49.6	162	49.1
Malaria endemicity				
High	102	27.8	88	26.7
Low	265	72.2	242	73.3
Exposure to artesunate interventions				
Case management training	87	23.7	66	20.0
Malaria guideline	118	32.2	131	39.7
Supportive supervision	39	10.6	29	8.8
Artesunate administration poster	224	61.0	157	47.6
Artesunate in stock	276	75.2	257	77.9

^a^ Denominator excludes 2 health workers with missing information in faith-based hospitals

^b^ Denominator excludes 3 health workers with missing information in government hospitals

### Knowledge of artesunate-based severe malaria treatment recommendations

Table 3 shows the correctness of health workers’ knowledge about artesunate-based severe malaria treatment recommendations stratified by hospital ownership and knowledge categories. Among respective government and FBO health workers, about a third of health workers had high knowledge of artesunate treatment policies (30.8% vs 32.9%), a third knew all dosing intervals (33.5% vs 33.3%), about half knew preparation solutions (49.9% vs 55.8%), half to two-thirds knew artesunate dose for both weight categories (50.8% vs 66.7%) and over three-quarters knew the preferred route of administration (78.7% vs 82.4%).

**Table 3:** Health workers’ knowledge about artesunate-based severe malaria treatment recommendations

Health workers’ knowledge	Government hospitals		Faith based hospitals	
	N = 367		N = 330	
	n	%	n	%
Treatment policy for severe malaria^a^				
High	113	30.8	108	32.9
Medium	131	35.7	101	30.8
Low	123	33.5	119	36.3
Artesunate dose^b^				
High	186	50.8	220	66.7
Medium	100	27.3	67	20.3
Low	80	21.9	43	13.0
Artesunate dosing intervals				
High	123	33.5	110	33.3
Medium	136	37.1	137	41.5
Low	108	29.4	83	25.2
Artesunate preparation				
High	183	49.9	184	55.8
Medium	134	36.5	114	34.5
Low	50	13.6	32	9.7
Preferred route of administration				
High	289	78.7	272	82.4
Low	78	21.3	58	17.6

^a^ Denominator excludes 2 health workers with missing information in faith-based hospitals

^b^ Denominator excludes 1 health worker with missing information in government hospitals

### Predictors of health workers knowledge about treatment recommendations

Tables in Additional files 1, 2, 3, 4, 5 show the results of univariable logistic regression analyses examining association between 11 factors and five knowledge outcomes about artesunate-based severe malaria treatment recommendations for each of the two hospital ownership sectors. In the government sector all 11 factors met the selection criteria of P < 0.15 for multivariable analyses on at least one knowledge outcome while in the FBO sector it was only health workers’ ward allocation that did not meet the same entrance criteria on any of the examined outcomes. For each of the knowledge outcomes, the multivariable results for the government and FBO hospitals are shown in Tables 4 and 5, respectively.

**Table 4:** Predictors of health workers knowledge about artesunate-based severe malaria treatment recommendations at government hospitals—multivariable models

Health workers’ knowledge on	Treatment policy			Artesunate dose			Artesunate dosing interval			Artesunate preparation			Artesunate route of administration		
	aOR	95% CI	P value	aOR	95% CI	P-value	aOR	95% CI	P-value	aOR	95% CI	P-value	aOR	95% CI	P-value
Age															
35–70 years				1.0 (ref)			1.0 (ref)								
21–35 years				1.70	0.96–3.01	0.071	1.19	0.71–2.02	0.509						
Gender															
Female	1.0 (ref)														
Male	1.17	0.74–1.85	0.497												
Cadre															
Nurse	1.0 (ref)			1.0 (ref)			1.0 (ref)			1.0 (ref)					
Clinician	1.86	1.18–2.91	0.007	1.14	0.66–1.95	0.645	1.23	0.75–2.05	0.412	0.80	0.53–1.20	0.272			
Ward															
Medical				1.0 (ref)			1.0 (ref)			1.0 (ref)			1.0 (ref)		
Paediatric				1.99	1.30–3.04	0.002	1.49	1.00–2.23	0.052	1.99	1.33–2.99	0.001	1.56	0.89–2.76	0.123
Endemicity															
Low													1.0 (ref)		
High													2.97	1.04–8.46	0.042
CM guidelines															
No				1.0 (ref)			1.0 (ref)						1.0 (ref)		
Yes				1.49	0.91–2.42	0.110	1.54	0.97–2.45	0.069				1.29	0.66–2.52	0.461
CM training															
No	1.0 (ref)									1.0 (ref)			1.0 (ref)		
Yes	2.31	1.44–3.72	0.001							1.58	0.96–2.60	0.071	1.95	0.86–4.39	0.108
Supervision															
No	1.0 (ref)						1.0 (ref)								
Yes	1.65	0.84–3.22	0.065				2.09	0.96–4.55	0.062						
AS poster															
No	1.0 (ref)			1.0 (ref)			1.0 (ref)			1.0 (ref)			1.0 (ref)		
Yes	1.21	0.78–1.90	0.398	2.17	1.24–3.79	0.007	1.43	0.85–2.42	0.179	1.40	0.88–2.22	0.154	2.19	1.05–4.57	0.037
AS in stock															
No				1.0 (ref)			1.0 (ref)			1.0 (ref)					
Yes				1.38	0.72–2.65	0.335	1.52	0.82–2.82	0.183	1.35	0.79–2.30	0.278			
Survey															
Baseline	1.0 (ref)			1.0 (ref)			1.0 (ref)			1.0 (ref)			1.0 (ref)		
Follow up	1.83	1.22–2.74	0.004	2.01	1.28–3.16	0.002	1.55	1.02–2.37	0.042	1.30	0.85–1.98	0.221	1.20	0.66–2.20	0.549

*CM* case-management, *AS* artesunate

**Table 5:** Predictors of health workers knowledge about artesunate-based severe malaria treatment recommendations at faith based hospitals—multivariable models

Health workers’ knowledge on	Treatment policy			Artesunate dose			Artesunate dosing interval			Artesunate preparation			Artesunate route of administration		
	aOR	95% CI	P-value	aOR	95% CI	P-value	aOR	95% CI	P-value	aOR	95% CI	P-value	aOR	95% CI	P-value
Age (years)															
35–70										1.0 (ref)					
21–35										0.70	0.36–1.31	0.260			
Gender															
Female	1.0 (ref)									1.0 (ref)					
Male	1.02	0.63–1.65	0.941							0.67	0.42–1.06	0.090			
Cadre															
Nurse	1.0 (ref)			1.0 (ref)			1.0 (ref)								
Clinician	2.27	1.41–3.65	0.001	2.24	1.33–3.77	0.002	1.76	1.15–2.69	0.009						
Endemicity															
Low													1.0 (ref)		
High													5.79	1.17–28.67	0.031
CM guidelines															
No	1.0 (ref)						1.0 (ref)								
Yes	2.41	1.48–3.93	<0.001				1.59	0.99–2.55	0.054						
CM training															
No	1.0 (ref)						1.0 (ref)			1.0 (ref)					
Yes	1.33	0.74–2.39	0.335				1.24	0.71–2.16	0.457	1.82	0.99–3.35	0.056			
Supervision															
No				1.0 (ref)			1.0 (ref)								
Yes				3.37	0.94–12.0)	0.061	1.42	0.64–3.16	0.391						
AS poster															
No				1.0 (ref)			1.0 (ref)			1.0 (ref)					
Yes	1.07	0.59–1.95	0.829	2.02	0.93–4.37	0.074	1.49	0.86–2.60	0.154	1.67	0.93–2.99	0.087			
AS in stock															
No	1.0 (ref)						1.0 (ref)			1.0 (ref)			1.0 (ref)		
Yes	2.01	1.05–3.85	0.036				2.18	1.20–3.94	0.010	1.61	0.88–2.96	0.121	4.73	1.50–14.89	0.008
Survey															
Baseline	1.0 (ref)			1.0 (ref)			1.0 (ref)			1.0 (ref)					
Follow up	1.61	0.94–2.77	0.085	1.31	0.70–2.45	0.392	1.09	0.66–1.82	0.733	1.38	0.81–2.36	0.240			

*CM* case management, *AS* artesunate

With respect to the treatment policy knowledge, clinicians compared to nurses were more likely to have high knowledge, both at the government (aOR = 1.86; 95% CI 1.18–2.91) and FBO hospitals (aOR = 2.27; 95% CI 1.41–3.65). In the government hospitals, health worker’s treatment policy knowledge was also significantly associated with training exposure (aOR = 2.31; 95% CI 1.44–3.72) and follow-up surveys (aOR = 1.83; 95% CI 1.22–2.74) while at the FBO hospitals the artesunate availability (aOR = 2.01; 95% CI 1.05–3.85) and access to guidelines (aOR = 2.41; 95% CI 1.48–3.93) were significant predictors.

Health workers’ knowledge about recommended artesunate dosing was significantly associated with displayed artesunate administration posters (aOR = 2.17; 95% CI 1.24–3.79), among paediatric compared to medical ward health workers (aOR = 1.99; 95% CI 1.30–3.04) and during the follow-up compared to the baseline survey (aOR = 2.01; 95% CI 1.28–3.16) within government hospitals. At the FBO hospitals, only health workers’ cadre was significant where clinicians were more likely to have correct dosing knowledge than nurses (aOR = 2.24; 95% CI 1.33–3.77). Regarding the knowledge of artesunate dosing intervals, the availability of artesunate (aOR = 2.18; 95% CI 1.20–3.94) and health workers’ cadre (aOR: 1.76; 95% CI 1.15–2.69) were significantly associated at the FBO hospitals while at the government hospitals the only significant predictor was the follow up compared to baseline survey (aOR = 1.55; 95% CI  1.02–2.37).

The knowledge of preferred artesunate administration route via intravenous slow bolus was significantly higher in high compared to low malaria risk areas, both among government (aOR = 2.97; 95% CI 1.04–8.46) and FBO health workers (aOR = 5.79; 95% CI 1.17–28.67). Furthermore, the same knowledge outcome was associated with displayed artesunate posters (aOR = 2.19; 95% CI 1.05–4.57) in the government hospitals and the artesunate availability (aOR = 4.73; 95% CI 1.50–14.89) in FBO hospitals. Finally, only one significant predictor, paediatric compared to medical ward allocation (aOR = 1.99; 95% CI 1.33–2.99) at the government hospitals was associated with the knowledge about artesunate preparation.

## Discussion

Five years following the change of national policy in Kenya, the knowledge of inpatient health workers about artesunate-based severe malaria treatment recommendations was suboptimal, both at the government and FBO hospitals. The analysis examining 11 factors of five knowledge outcomes in the two hospital sectors reveals several patterns of importance for artesunate policy implementers in Kenya and other African countries.

In-service training, commonly used intervention for knowledge translation, was found to be associated with higher knowledge about the new severe malaria treatment policy among government health workers, however, without an effect on the subtler artesunate treatment standards, such as correct knowledge about dosing, dosing intervals, drug preparation and preferred route of administration. A similar pattern was revealed at the FBO hospitals with respect to health workers’ exposure to malaria guidelines. Lack of training and guideline association with health workers’ knowledge has been reported in Kenya [16], elsewhere in Africa [17–19] and limited beneficial effects observed in this study may reflect the short time allocated to severe malaria topic (only 2–3 h) within the 3-day malaria case management training curriculum, lack of guideline suitability to transfer more subtle knowledge information, or eventually suboptimal quality of implementation when interventions, such as in-service training, are delivered programmatically on a large, national scale [19, 20].

In contrast to training and access to guidelines, the correct dosing knowledge was associated with simple job aids such as displayed posters focusing on recommended artesunate dosing, preparation and administration within the wards. The results concur with the study showing the effects of poster reminders on health workers surveillance knowledge in Kenya [16]. Interestingly, the effects of such job-aids are observed within the government sector while at the FBO hospitals commodity availability seems to be a prevailing contextual determinant, both of the treatment policy and artesunate use knowledge. Lack of access to subsidized artesunate by FBO hospitals may be a factor underlying knowledge deficiencies at the hospitals where absence of artesunate is unlikely due to experienced stock-outs, but more likely the result of failed implementation due to high cost of artesunate.

Clinicians compared to nurses had higher knowledge levels. The pre-service training effect, given the non-prescribing role of nurses in the inpatient setting, was expectedly marked regarding the drug policy knowledge and correct dosing. Higher artesunate preparation knowledge was however not found among nurses, the cadre routinely performing this task in the hospital inpatient setting. This was not also observed in Tanzania where artesunate preparation knowledge was higher among clinicians compared to nurses [13]. What has been however observed in the study reported in this manuscript is higher artesunate preparation and dosing knowledge among paediatric ward health workers at the government hospitals, the findings likely reflecting greater traditional focus towards paediatric malaria care [21, 22]. Future interventions should pay greater attention to the health workers managing adult patients in the medical wards.

Follow up surveys at the government hospitals have been associated with correct knowledge about the treatment policy and recommended dosing despite small proportion of less than 5% of health workers being repeatedly interviewed. Provision of correct responses after the knowledge assessments, dissemination of national malaria guidelines, and display of artesunate posters including informal consultations between the interviewed health workers and the study personnel does suggest that surveys at the study hospitals may not present only measurement and monitoring activity, but also have an intervention effect per se.

Finally, few limitations should be mentioned. First, while knowledge about the new treatment policy does present basic pre-requisite for the drug policy implementation the results of this study do not reflect actual clinical practices. Second, while these results apply to major government and FBO hospitals nationally they do not reflect knowledge and determinants at smaller facilities with inpatient capacities. Finally, some of the significant associations may have not been observed due to power limitations while conversely multiple comparisons performed may have resulted in some of the associations being due to chance.

## Conclusions

Programmatic interventions ensuring display of artesunate administration posters in the wards, targeting of health workers managing adult patients in the medical wards and repeated knowledge assessments are likely to be beneficial for improving the knowledge of government health workers about artesunate-based severe malaria treatment recommendations. The availability of artesunate and focus on improvements of nurses’ knowledge should be prioritized at the FBO hospitals.

## Supplementary information

**Additional file 1.** Univariable ordinal logistic regression analysis of predictors of knowledge on severe malaria treatment policy, by hospital ownership.

**Additional file 2.** Univariable ordinal logistic regression analysis of predictors of artesunate dose knowledge, by hospital ownership.

**Additional file 3.** Univariable ordinal logistic regression analysis of predictors of artesunate dosing interval knowledge, by hospital ownership.

**Additional file 4.** Univariable ordinal logistic regression analysis of predictors of artesunate preparation knowledge, by hospital ownership.

**Additional file 5.** Univariable binary logistic regression analysis of predictors of the knowledge about preferred route of artesunate, by hospital ownership.

## Data Availability

The datasets used and analyzed during the current study are available from the corresponding author on reasonable request.

## Abbreviations

aOR: Adjusted odds ratio
CI: Confidence interval
FBO: Faith-based organization
WHO: World Health Organization
