# Supplementary material for: Predictors of health workers’ knowledge about artesunate-based severe malaria treatment recommendations in government and faith-based hospitals in Kenya
Source: Malar J. 2020 Jul 23;19:267. doi: 10.1186/s12936-020-03341-2 (PMC7379778; doi:10.1186/s12936-020-03341-2)
Supplement: Supplementary file 1 — Additional file 1. Univariable ordinal logistic regression analysis of predictors of knowledge on severe malaria treatment policy, by hospital ownership. [file 12936_2020_3341_MOESM1_ESM.docx]

**Additional file 1. Univariable ordinal logistic regression analysis of predictors of knowledge on severe malaria treatment policy, by hospital ownership**

|  | **GoK hospitals** | | | | | | **FBO hospitals** | | | | | |
| --- | --- | --- | --- | --- | --- | --- | --- | --- | --- | --- | --- | --- |
|  | **N** | **Low**  **n (%)** | **Medium**  **n (%)** | **High**  **n (%)** | **OR**  **(95% CI)** | **p-value** | **N** | **Low**  **n (%)** | **Medium**  **n (%)** | **High**  **n (%)** | **OR**  **(95% CI)** | **p-value** |
| **Age** |  |  |  |  |  |  |  |  |  |  |  |  |
| 35-70 years | 138 | 50(36.2) | 51(37.0) | 37(26.8) | 1.0(ref) |  | 57 | 23(40.4) | 15(26.3) | 19(33.3) | 1.0(ref) |  |
| 21-35 years | 229 | 73(31.9) | 80(34.9) | 76(33.2) | 1.29(0.87-1.92) | 0.205 | 269 | 96(35.7) | 85(31.6) | 88(32.7) | 1.10(0.62-1.93) | 0.750 |
| **Sex** |  |  |  |  |  |  |  |  |  |  |  |  |
| Female | 227 | 82(36.1) | 82(36.1) | 63(27.8) | 1.0(ref) |  | 168 | 69(41.1) | 50(29.8) | 49(29.2) | 1.0(ref) |  |
| Male | 140 | 41(29.3) | 49(35.0) | 50(35.7) | 1.45(0.97-2.16) | 0.072 | 160 | 50(31.3) | 51(31.9) | 59(36.9) | 1.58(1.03-2.43) | 0.034 |
| **Cadre** |  |  |  |  |  |  |  |  |  |  |  |  |
| Nurse | 192 | 78(40.6) | 65(33.9) | 49(25.5) | 1.0(ref) |  | 174 | 80(46.0) | 51(29.3) | 43(24.7) | 1.0(ref) |  |
| Clinician | 175 | 45(25.7) | 66(37.7) | 64(36.6) | 1.91(1.29-2.82) | 0.001 | 154 | 39(25.3) | 50(32.5) | 65(42.2) | 2.51(1.64-3.85) | <0.001 |
| **Ward** |  |  |  |  |  |  |  |  |  |  |  |  |
| Medical | 182 | 58(31.9) | 72(39.6) | 52(28.6) | 1.0(ref) |  | 162 | 60(37.0) | 55(34.0) | 47(29.0) | 1.0(ref) |  |
| Paediatric | 185 | 65(35.1) | 59(31.9) | 61(33.0) | 1.02(0.70-1.49) | 0.932 | 166 | 59(35.5) | 46(27.7) | 61(36.7) | 1.24(0.82-1.87) | 0.308 |
| **Endemicity** |  |  |  |  |  |  |  |  |  |  |  |  |
| Low | 265 | 90(34.0) | 91(34.3) | 84(31.7) | 1.0(ref) |  | 240 | 83(34.6) | 73(30.4) | 84(35.0) | 1.0(ref) |  |
| High | 102 | 33(32.4) | 40(39.2) | 29(28.4) | 0.95(0.59-1.55) | 0.848 | 88 | 36(40.9) | 28(31.8) | 24(27.3) | 0.70(0.37-1.33) | 0.274 |
| **CM Guidelines** |  |  |  |  |  |  |  |  |  |  |  |  |
| No | 249 | 85(34.1) | 92(36.9) | 72(28.9) | 1.0(ref) |  | 198 | 92(46.5) | 54(27.3) | 52(26.3) | 1.0(ref) |  |
| Yes | 118 | 38(32.2) | 39(33.1) | 41(34.7) | 1.17(0.78-1.78) | 0.447 | 129 | 27(20.9) | 46(35.7) | 56(43.4 | 2.89(1.81-4.61) | 0.000 |
| **CM training** |  |  |  |  |  |  |  |  |  |  |  |  |
| No | 280 | 108(38.6) | 93(33.2) | 79(28.2) | 1.0(ref) |  | 263 | 105(39.9) | 77(29.3) | 81(30.8) | 1.0(ref) |  |
| Yes | 87 | 15(17.2) | 38(43.7) | 34(39.1) | 2.09(1.33-3.30) | 0.002 | 65 | 14(21.5) | 24(36.9) | 27(41.5)5) | 1.83(1.07-3.14) | 0.027 |
| **Supervision** |  |  |  |  |  |  |  |  |  |  |  |  |
| No | 328 | 117(35.7) | 115(35.1) | 96(29.3) | 1.0(ref) |  | 299 | 109(36.5) | 92(30.8) | 98(32.8) | 1.0(ref) |  |
| Yes | 39 | 6(15.4) | 16(41.0) | 17(43.6) | 2.21(1.18-4.16) | 0.014 | 29 | 10(34.5) | 9(31.0) | 10(34.5) | 1.46(0.65-3.26) | 0.359 |
| **AS poster** |  |  |  |  |  |  |  |  |  |  |  |  |
| **No** | 143 | 55(38.5) | 51(35.7) | 37(25.9) | 1.0(ref) |  | 171 | 65(38.0) | 60(35.1) | 46(26.9) | 1.0(ref) |  |
| Yes | 224 | 68(30.4) | 80(35.7) | 76(33.9) | 1.44(0.95-2.18) | 0.084 | 157 | 54(34.4) | 41(26.1) | 62(39.5) | 1.44(0.91-2.28) | 0.122 |
| **AS in stock** |  |  |  |  |  |  |  |  |  |  |  |  |
| No | 91 | 30(33.0) | 39(42.9) | 22(24,2) | 1.0(ref) |  | 73 | 37(50.7) | 18(24.7) | 18(24.7) | 1.0(ref) |  |
| Yes | 276 | 93(33.7) | 92(33.3) | 91(33.0) | 1.27(0.78-2.07) | 0.337 | 255 | 82(32.2) | 83(32.5) | 90(35.3) | 2.01(1.08-3.73) | 0.028 |
| **Survey** |  |  |  |  |  |  |  |  |  |  |  |  |
| Baseline | 185 | 72(38.9) | 70(37.8) | 43(23.2) | 1.0(ref) |  | 163 | 68(41.7) | 51(31.3) | 44(27) | 1.0(ref) |  |
| Follow up | 182 | 51(28) | 61(33.5) | 70(38.5) | 1.89(1.28-2.78) | 0.001 | 165 | 51(30.9) | 50(30.3) | 64(38.8) | 1.74(1.15-2.63) | 0.009 |
