# Supplementary material for: Predictors of health workers’ knowledge about artesunate-based severe malaria treatment recommendations in government and faith-based hospitals in Kenya
Source: Malar J. 2020 Jul 23;19:267. doi: 10.1186/s12936-020-03341-2 (PMC7379778; doi:10.1186/s12936-020-03341-2)
Supplement: Supplementary file 2 — Additional file 2. Univariable ordinal logistic regression analysis of predictors of artesunate dose knowledge, by hospital ownership. [file 12936_2020_3341_MOESM2_ESM.docx]

**Additional file 2. Univariable ordinal logistic regression analysis of predictors of artesunate dose knowledge, by hospital ownership**

|  | **GoK hospitals** | | | | | | **FBO hospitals** | | | | | |
| --- | --- | --- | --- | --- | --- | --- | --- | --- | --- | --- | --- | --- |
|  | **N** | **Low**  **n (%)** | **Medium**  **n (%)** | **High**  **n (%)** | **OR**  **(95% CI)** | **p-value** | **N** | **Low**  **n (%)** | **Medium**  **n (%)** | **High**  **n (%)** | **OR**  **(95% CI)** | **p-value** |
| **Age** |  |  |  |  |  |  |  |  |  |  |  |  |
| 35-70 years | 137 | 38(27.7) | 38(27.7) | 61(44.5) | 1.0(ref) |  | 57 | 9(15.8) | 12(21.1) | 36(63.2) | 1.0(ref) |  |
| 21-35 years | 229 | 42(18.3) | 62(27.1) | 125(54.6) | 1.77(1.14-2.73) | 0.010 | 271 | 34(12.5) | 55(20.3) | 18267.2) | 1.41(0.73-2.72) | 0.309 |
| **Sex** |  |  |  |  |  |  |  |  |  |  |  |  |
| Female | 226 | 58(25.7) | 54(23.9) | 114(50.4) | 1.0(ref) |  | 169 | 24(14.2) | 38(22.5) | 107(63.3) | 1.0(ref) |  |
| Male | 140 | 22(15.7) | 46(32.9) | 72(51.4) | 1.37(0.88-2.11) | 0.162 | 161 | 19(11.8) | 29(18.0) | 113(70.2) | 1.23(0.74-2.05) | 0.421 |
| **Cadre** |  |  |  |  |  |  |  |  |  |  |  |  |
| Nurse | 191 | 48(25.1) | 53(27.7) | 90(47.1) | 1.0(ref) |  | 174 | 33(19.0) | 36(20.7) | 105(60.3) | 1.0(ref) |  |
| Clinician | 175 | 32(18.3) | 47(26.9) | 96(54.9) | 1.45(0.96-2.18) | 0.078 | 156 | 10(6.4) | 31(19.9) | 115(73.7) | 2.21(1.33-3.67) | 0.002 |
| **Ward** |  |  |  |  |  |  |  |  |  |  |  |  |
| Medical | 181 | 43(23.8) | 63(34.8) | 75(41.4) | 1.0(ref) |  | 162 | 21(13.0) | 33(20.4) | 108(66.7) | 1.0(ref) |  |
| Paediatric | 185 | 37(20.0) | 37(20.0) | 111(60.0) | 1.94(1.29-2.94) | 0.002 | 168 | 22(13.1) | 34(20.2) | 112(66.7) | 0.94(0.58-1.53) | 0.815 |
| **Endemicity** |  |  |  |  |  |  |  |  |  |  |  |  |
| Low | 264 | 61(23.1) | 72(27.3) | 131(49.6) | 1.0(ref) |  | 242 | 36(14.9) | 44(18.2) | 162(66.9) | 1.0(ref) |  |
| High | 102 | 19(18.6) | 28(27.5) | 55(53.9) | 1.25(0.63-2.48) | 0.521 | 88 | 7(8.0) | 23(26.1) | 58(65.9) | 1.08(0.44-2.68) | 0.868 |
| **CM Guidelines** |  |  |  |  |  |  |  |  |  |  |  |  |
| No | 249 | 63(25.3) | 66(26.5) | 120(48.2) | 1.0(ref) |  | 198 | 32(16.2) | 43(21.7) | 123(62.1) | 1.0(ref) |  |
| Yes | 117 | 17(14.5) | 34(29.1) | 66(56.4) | 1.76(1.11-2.80) | 0.016 | 131 | 11(8.4) | 24(18.3) | 96(73.3) | 1.50(0.86-2.61) | 0.151 |
| **CM training** |  |  |  |  |  |  |  |  |  |  |  |  |
| No | 279 | 61(21.9) | 84(30.1) | 134(48.0) | 1.0(ref) |  | 264 | 38(14.4) | 53(20.1) | 173(65.5) | 1.0(ref) |  |
| Yes | 87 | 19(21.8) | 16(18.4) | 52(59.8) | 1.41(0.83-2.39) | 0.198 | 66 | 5(7.6) | 14(21.2) | 47(71.2) | 1.17(0.61-2.26) | 0.640 |
| **Supervision** |  |  |  |  |  |  |  |  |  |  |  |  |
| No | 327 | 76(23.2) | 86(26.3) | 165(50.5) | 1.0(ref) |  | 301 | 43(14.3) | 63(20.9) | 195(64.8) | 1.0(ref) |  |
| Yes | 39 | 4(10.3) | 14(35.9) | 21(53.8) | 1.13(0.56-2.26) | 0.739 | 29 | 0(0.0) | 4(13.8) | 25(86.2) | 4.15(1.24-13-92) | 0.021 |
| **AS poster** |  |  |  |  |  |  |  |  |  |  |  |  |
| **No** | 143 | 43(30.1) | 48(33.6) | 52(36.4) | 1.0(ref) |  | 173 | 27(15.6) | 45(26.0) | 101(58.4) | 1.0(ref) |  |
| Yes | 223 | 37(16.6) | 52(23.3) | 134(60.1) | 2.92(1.75-4.86) | <0.001 | 157 | 16(10.2) | 22(14.0) | 119(75.8) | 2.57(1.40-4.73) | 0.002 |
| **AS in stock** |  |  |  |  |  |  |  |  |  |  |  |  |
| No | 91 | 30(33.0) | 20(22.0) | 41(45.1) | 1.0(ref) |  | 73 | 15(20.5) | 16(21.9) | 42(57.5) | 1.0(ref) |  |
| Yes | 275 | 50(18.2) | 80(29.1) | 145(52.7) | 2.13(1.15-3.92) | 0.015 | 257 | 28(10.9) | 51(19.8) | 178(69.3) | 1.71(0.80-3.68) | 0.167 |
| **Survey** |  |  |  |  |  |  |  |  |  |  |  |  |
| Baseline | 185 | 50(27.0) | 61(33.0) | 74(40.0) | 1.0(ref) |  | 164 | 24(14.6) | 42(25.6) | 98(59.8) | 1.0(ref) |  |
| Follow up | 181 | 30(16.6) | 39(21.5) | 112(61.9) | 2.41(1.59-3.67) | 0.000 | 166 | 19(11.4) | 25(15.1) | 122(73.5) | 1.91(1.17-3.12) | 0.010 |
