# Supplementary material for: Predictors of health workers’ knowledge about artesunate-based severe malaria treatment recommendations in government and faith-based hospitals in Kenya
Source: Malar J. 2020 Jul 23;19:267. doi: 10.1186/s12936-020-03341-2 (PMC7379778; doi:10.1186/s12936-020-03341-2)
Supplement: Supplementary file 3 — Additional file 3. Univariable ordinal logistic regression analysis of predictors of artesunate dosing interval knowledge, by hospital ownership. [file 12936_2020_3341_MOESM3_ESM.docx]

**Additional file 3. Univariable ordinal logistic regression analysis of predictors of artesunate dosing interval knowledge, by hospital ownership**

|  | **GoK hospitals** | | | | | | **FBO hospitals** | | | | | |
| --- | --- | --- | --- | --- | --- | --- | --- | --- | --- | --- | --- | --- |
|  | **N** | **Low**  **n (%)** | **Medium**  **n (%)** | **High**  **n (%)** | **OR**  **(95% CI)** | **p-value** | **N** | **Low**  **n (%)** | **Medium**  **n (%)** | **High**  **n (%)** | **OR**  **(95% CI)** | **p-value** |
| **Age** |  |  |  |  |  |  |  |  |  |  |  |  |
| 35-70 years | 138 | 42(30.4) | 58(42.0) | 38(27.5) | 1.0(ref) |  | 57 | 14(24.6) | 26(45.6) | 17(29.8) | 1.0(ref) |  |
| 21-35 years | 229 | 66(28.8) | 78(34.1) | 85(37.1) | 1.48(0.97-2.25) | 0.067 | 271 | 69(25.5) | 109(40.2) | 93(34.3) | 1.05(0.61-1.83) | 0.854 |
| **Sex** |  |  |  |  |  |  |  |  |  |  |  |  |
| Female | 227 | 70(30.8) | 83(36.6) | 74(32.6) | 1.0(ref) |  | 169 | 44(26.0) | 76(45.0) | 49(29.0) | 1.0(ref) |  |
| Male | 140 | 38(27.1) | 53(37.9) | 49(35.0) | 1.31(0.86-1.99) | 0.211 | 161 | 39(24.2) | 61(37.9) | 61(37.9) | 1.35(0.88-2.06) | 0.172 |
| **Cadre** |  |  |  |  |  |  |  |  |  |  |  |  |
| Nurse | 192 | 59(30.7) | 81(42.2) | 52(27.1) | 1.0(ref) |  | 174 | 53(30.5) | 73(42.0) | 48(27.6) | 1.0(ref) |  |
| Clinician | 175 | 49(28.0) | 55(31.4) | 71(40.6) | 1.50(1.01-2.23) | 0.046 | 156 | 30(19.2) | 64(41.0) | 62(39.7) | 1.90(1.25-2.90) | 0.003 |
| **Ward** |  |  |  |  |  |  |  |  |  |  |  |  |
| Medical | 182 | 58(31.9) | 70(38.5) | 54(29.7) | 1.0(ref) |  | 162 | 41(25.3) | 66(40.7) | 55(34.0) | 1.0(ref) |  |
| Paediatric | 185 | 50(27.0) | 66(35.7) | 69(37.3) | 1.44(0.97-2.13) | 0.074 | 168 | 42(25.0) | 71(42.3) | 55(32.7) | 0.96(0.64-1.45) | 0.863 |
| **Endemicity** |  |  |  |  |  |  |  |  |  |  |  |  |
| Low | 265 | 87(32.8) | 91(34.3) | 87(32.8) | 1.0(ref) |  | 242 | 65(26.9) | 97(40.1) | 80(33.1) | 1.0(ref) |  |
| High | 102 | 21(20.6) | 45(44.1) | 36(35.3) | 1.49(0.76-2.94) | 0.250 | 88 | 18(20.5) | 40(45.5) | 30(34.1) | 1.20(0.65-2.21) | 0.566 |
| **CM Guidelines** |  |  |  |  |  |  |  |  |  |  |  |  |
| No | 249 | 75(30.1) | 101(40.6) | 73(29.3) | 1.0(ref) |  | 198 | 60(30.3) | 80(40.4) | 58(29.3) | 1.0(ref) |  |
| Yes | 118 | 33(28.0) | 35(29.7) | 50(42.4) | 1.79(1.14-2.82) | 0.012 | 131 | 23(17.6) | 56(42.7) | 52(39.7) | 1.85(1.17-2.91) | 0.008 |
| **CM training** |  |  |  |  |  |  |  |  |  |  |  |  |
| No | 280 | 82(29.3) | 102(36.4) | 96(34.3) | 1.0(ref) |  | 264 | 73(27.7) | 106(40.2) | 85(32.2) | 1.0(ref) |  |
| Yes | 87 | 26(29.9) | 34(39.1) | 27(31.0) | 0.96(0.59-1.56) | 0.864 | 66 | 10(15.2) | 31(47.0) | 25(37.9) | 1.49(0.88-2.52) | 0.142 |
| **Supervision** |  |  |  |  |  |  |  |  |  |  |  |  |
| No | 328 | 100(30.5) | 128(39.0) | 100(30.5) | 1.0(ref) |  | 301 | 77(25.6) | 127(42.2) | 97(32.2) | 1.0(ref) |  |
| Yes | 39 | 8(20.5) | 8(20.5) | 23(59.0) | 2.55(1.23-5.29) | 0.012 | 29 | 6(20.7) | 10(34.5) | 13((44.8) | 1.82(0.82-4.03) | 0.142 |
| **AS poster** |  |  |  |  |  |  |  |  |  |  |  |  |
| **No** | 143 | 52(36.4) | 56(39.2) | 35(24.5) | 1.0(ref) |  | 173 | 48(27.7) | 78(45.1) | 47(27.2) | 1.0(ref) |  |
| Yes | 224 | 56(25.0) | 80(35.7) | 88(39.3) | 1.94(1.19-3.17) | 0.008 | 157 | 35(22.3) | 59(37.6) | 63(40.1) | 1.59(1.01-2.51) | 0.047 |
| **AS in stock** |  |  |  |  |  |  |  |  |  |  |  |  |
| No | 91 | 37(40.7) | 33(36.3) | 21(23.1) | 1.0(ref) |  | 73 | 29(39.7) | 26(35.6) | 18(24.7) | 1.0(ref) |  |
| Yes | 276 | 71(25.7) | 103(37.3) | 102(37.0) | 2.17(1.22-3.86) | 0.008 | 257 | 54(21.0) | 111(43.2) | 92(35.8) | 2.02(1.12-3.65) | 0.020 |
| **Survey** |  |  |  |  |  |  |  |  |  |  |  |  |
| Baseline | 185 | 61(33.0) | 78(42.2) | 46(24.9) | 1.0(ref) |  | 164 | 46(28.0) | 70(42.7) | 48(29.3) | 1.0(ref) |  |
| Follow up | 182 | 47(25.8) | 58(31.9) | 77(42.3) | 1.85(0.86-2.76) | 0.002 | 166 | 37(22.3) | 67(40.4) | 62(37.3) | 1.47(0.97-2.21) | 0.067 |
