# Supplementary material for: Predictors of health workers’ knowledge about artesunate-based severe malaria treatment recommendations in government and faith-based hospitals in Kenya
Source: Malar J. 2020 Jul 23;19:267. doi: 10.1186/s12936-020-03341-2 (PMC7379778; doi:10.1186/s12936-020-03341-2)
Supplement: Supplementary file 4 — Additional file 4. Univariable ordinal logistic regression analysis of predictors of artesunate preparation knowledge, by hospital ownership. [file 12936_2020_3341_MOESM4_ESM.docx]

**Additional file 4. Univariable ordinal logistic regression analysis of predictors of artesunate preparation knowledge, by hospital ownership**

|  | **GoK hospitals** | | | | | | **FBO hospitals** | | | | | |
| --- | --- | --- | --- | --- | --- | --- | --- | --- | --- | --- | --- | --- |
|  | **N** | **Low**  **n (%)** | **Medium**  **n (%)** | **High**  **n (%)** | **OR**  **(95% CI)** | **p-value** | **N** | **Low**  **n (%)** | **Medium**  **n (%)** | **High**  **n (%)** | **OR**  **(95% CI)** | **p-value** |
| **Age** |  |  |  |  |  |  |  |  |  |  |  |  |
| 35-70 years | 138 | 21(15.2) | 44(31.9) | 73(52.9) | 1.0(ref) |  | 57 | 5(8.8) | 14(24.6) | 38(66.7) | 1.0(ref) |  |
| 21-35 years | 229 | 29(12.7) | 90(39.3) | 110(48.0) | 0.89(0.59-1.34) | 0.570 | 271 | 26(9.6) | 100(36.9) | 145(53.5) | 0.60(0.32-1.11) | 0.103 |
| **Sex** |  |  |  |  |  |  |  |  |  |  |  |  |
| Female | 227 | 34(15.0) | 83(36.6) | 110(48.5) | 1.0(ref) |  | 169 | 13(7.7) | 56(33.1) | 100(59.2) | 1.0(ref) |  |
| Male | 140 | 16(11.4) | 51(36.4) | 73(52.1) | 1.20(0.80-1.82) | 0.377 | 161 | 19(11.8) | 58(36.0) | 84(52.2) | 0.70(0.45-1.10) | 0.120 |
| **Cadre** |  |  |  |  |  |  |  |  |  |  |  |  |
| Nurse | 192 | 26(13.5) | 62(32.3) | 104(54.2) | 1.0(ref) |  | 174 | 19(10.9) | 53(30.5) | 102(58.6) | 1.0(ref) |  |
| Clinician | 175 | 24(13.7) | 72(41.1) | 79(45.1) | 0.74(0.50-1.10) | 0.142 | 156 | 13(8.3) | 61(39.1) | 82(52.6) | 0.87(0.56-1.34) | 0.520 |
| **Ward** |  |  |  |  |  |  |  |  |  |  |  |  |
| Medical | 182 | 31(17.0) | 76(41.8) | 75(41.2) | 1.0(ref) |  | 162 | 17(10.5) | 56((34.6) | 89(54.9) | 1.0(ref) |  |
| Paediatric | 185 | 19(10.3) | 58(31.4) | 108(58.4) | 2.00(1.34-2.99) | 0.001 | 168 | 15(8.9) | 58(34.5) | 95(56.5) | 1.10(0.71-1.69) | 0.671 |
| **Endemicity** |  |  |  |  |  |  |  |  |  |  |  |  |
| Low | 265 | 37(14.0) | 95(35.8) | 133(50.2) | 1.0(ref) |  | 242 | 24(9.9) | 79(32.6) | 139(57.4) | 1.0(ref) |  |
| High | 102 | 13(12.7) | 39(38.2) | 50(49.0) | 1.00(0.61-1.64) | 0.985 | 88 | 8(9.1) | 35(39.8) | 45(51.1) | 0.82(0.46-1.45) | 0.493 |
| **CM Guidelines** |  |  |  |  |  |  |  |  |  |  |  |  |
| No | 249 | 35(14.1) | 97(39.0) | 117(47 | 1.0(ref) |  | 198 | 20(10.1) | 73(36.9) | 105(53.0) | 1.0(ref) |  |
| Yes | 118 | 15(12.7) | 37(31.40 | 66(5.9) | 1.37(0.88-2.11) | 0.157 | 131 | 11(8.4) | 41(31.3) | 79(60.3) | 1.33(0.84-2.12) | 0.229 |
| **CM training** |  |  |  |  |  |  |  |  |  |  |  |  |
| No | 280 | 43(15.4) | 105(37.5) | 132(47.1) | 1.0(ref) |  | 264 | 28(10.6) | 95(36.0) | 141(53.4) | 1.0(ref) |  |
| Yes | 87 | 7(8.0) | 29(33.3) | 51(58.6) | 1.66(1.02-2.70) | 0.041 | 66 | 4(6.1) | 19(28.8) | 43(65.2) | 1.80(1.00-3.24) | 0.049 |
| **Supervision** |  |  |  |  |  |  |  |  |  |  |  |  |
| No | 328 | 45(13.7) | 119(36.3) | 164(50.0) | 1.0(ref) |  | 301 | 31(10.3) | 103(34.2) | 167(55.5) | 1.0(ref) |  |
| Yes | 39 | 5(12.8) | 15(38.5) | 19(48.7) | 0.95(0.50-1.83) | 0.888 | 29 | 1(3.4) | 11(37.9) | 17(58.6) | 1.33(0.60-2.96) | 0.481 |
| **AS poster** |  |  |  |  |  |  |  |  |  |  |  |  |
| **No** | 143 | 31(21.7) | 46(32.2) | 66(46.2) | 1.0(ref) |  | 173 | 19(11.0) | 72(41.6) | 82(47.4) | 1.0(ref) |  |
| Yes | 224 | 19(8.5) | 88(39.3) | 117(52.2) | 1.56(1.01-2.39) | 0.043 | 157 | 13(8.3) | 42(26.8) | 102(65.0) | 1.97(1.24-3.13) | 0.004 |
| **AS in stock** |  |  |  |  |  |  |  |  |  |  |  |  |
| No | 91 | 17(18.7) | 35(38.5) | 39(42.9) | 1.0(ref) |  | 73 | 12(16.4) | 25(34.2) | 36(49.3) | 1.0(ref) |  |
| Yes | 276 | 33(12.0) | 99(35.9) | 144(52.2) | 1.65(0.98-2.76) | 0.058 | 257 | 20(7.8) | 89(34.6) | 148(57.6) | 1.61(0.90-2.88) | 0.110 |
| **Survey** |  |  |  |  |  |  |  |  |  |  |  |  |
| Baseline | 185 | 29(15.7) | 73(39.5) | 83(44.9) | 1.0(ref) |  | 164 | 19(11.6) | 65(39.6) | 80(48.8) | 1.0(ref) |  |
| Follow up | 182 | 21(11.5) | 61(33.5) | 100(54.9) | 1.49(1.00-2.21) | 0.049 | 166 | 13(7.8) | 49(29.5) | 104(62.7) | 1.78(1.15-2.76) | 0.009 |
