# Supplementary material for: Predictors of health workers’ knowledge about artesunate-based severe malaria treatment recommendations in government and faith-based hospitals in Kenya
Source: Malar J. 2020 Jul 23;19:267. doi: 10.1186/s12936-020-03341-2 (PMC7379778; doi:10.1186/s12936-020-03341-2)
Supplement: Supplementary file 5 — Additional file 5. Univariable binary logistic regression analysis of predictors of the knowledge about preferred route of artesunate, by hospital ownership. [file 12936_2020_3341_MOESM5_ESM.docx]

**Additional file 5. Univariable binary logistic regression analysis of predictors of the knowledge about preferred route of artesunate, by hospital ownership**

|  | **GoK hospitals** | | | | | **FBO hospitals** | | | | |
| --- | --- | --- | --- | --- | --- | --- | --- | --- | --- | --- |
|  | **N** | **Low**  **n (%)** | **High**  **n (%)** | **OR**  **(95% CI)** | **p-value** | **N** | **Low**  **n (%)** | **High**  **n (%)** | **OR**  **(95% CI)** | **p-value** |
| **Age** |  |  |  |  |  |  |  |  |  |  |
| 35-70 years | 138 | 31(22.5) | 107(77.5) | 1.0(ref) |  | 57 | 14(24.6) | 43(75.4) | 1.0(ref) |  |
| 21-35 years | 229 | 47(20.5) | 182(79.5) | 1.11(0.62-1.99) | 0.735 | 271 | 44(16.2) | 227(83.8) | 1.55(0.67-3.61) | 0.309 |
| **Sex** |  |  |  |  |  |  |  |  |  |  |
| Female | 227 | 52(22.9) | 175(77.1) | 1.0(ref) |  | 169 | 33(19.5) | 136(80.5) | 1.0(ref) |  |
| Male | 140 | 26(18.6) | 114(81.4) | 1.30(0.71-2.38) | 0.398 | 161 | 25(15.5) | 136(84.5) | 1.11(0.55-2.22) | 0.767 |
| **Cadre** |  |  |  |  |  |  |  |  |  |  |
| Nurse | 192 | 39(20.3) | 153(79.7) | 1.0(ref) |  | 174 | 29(16.7) | 145(83.3) | 1.0(ref) |  |
| Clinician | 175 | 39(22.3) | 136(77.7) | 0.89(0.51-1.55) | 0.675 | 156 | 29(18.6) | 127(81.4) | 0.91(0.47-1.77) | 0.779 |
| **Ward** |  |  |  |  |  |  |  |  |  |  |
| Medical | 182 | 44(24.2) | 138(75.5) | 1.0(ref) |  | 162 | 26(16.0) | 136(84.0) | 1.0(ref) |  |
| Paediatric | 185 | 34(18.4) | 151(81.6) | 1.56(0.89-2.73) | 0.120 | 168 | 32(19.0) | 136(81.0) | 0.68(0.34-1.33) | 0.259 |
| **Endemicity** |  |  |  |  |  |  |  |  |  |  |
| Low | 265 | 66(24.9) | 199(75.1) | 1.0(ref) |  | 242 | 53(21.9) | 189(78.1) | 1.0(ref) |  |
| High | 102 | 12(11.8) | 90(88.2) | 2.97(1.03-8.56) | 0.044 | 88 | 5(5.7) | 83(94.3) | 6.86(1.28-36.79) | 0.025 |
| **CM Guidelines** |  |  |  |  |  |  |  |  |  |  |
| No | 249 | 58(23.3) | 191(76.7) | 1.0(ref) |  | 198 | 36(18.2) | 162(81.8) | 1.0(ref) |  |
| Yes | 118 | 20(16.9) | 98(83.1) | 1.68(0.88-3.22) | 0.117 | 131 | 22(16.8) | 109(83.2) | 0.77(0.35-1.69) | 0.512 |
| **CM training** |  |  |  |  |  |  |  |  |  |  |
| No | 280 | 67(23.9) | 213(76.1) | 1.0(ref) |  | 264 | 47(17.8) | 217(82.2) | 1.0(ref) |  |
| Yes | 87 | 11(12.6) | 76(87.4) | 1.99(0.91-4.36) | 0.086 | 66 | 11(16.7) | 55(83.3) | 1.18(0.48-2.91) | 0.723 |
| **Supervision** |  |  |  |  |  |  |  |  |  |  |
| No | 328 | 73(22.3) | 255(77.7) | 1.0(ref) |  | 301 | 54(17.9) | 247(82.1) | 1.0(ref) |  |
| Yes | 39 | 5(12.8) | 34(87.2) | 1.57(0.52-4.72) | 0.421 | 29 | 4(13.8) | 25(86.2) | 0.99(0.24-4.09) | 0.986 |
| **AS poster** |  |  |  |  |  |  |  |  |  |  |
| **No** | 143 | 42(29.4) | 101(70.6) | 1.0(ref) |  | 173 | 35(20.2) | 138(79.8) | 1.0(ref) |  |
| Yes | 224 | 36(16.1 | 188(83.9 | 2.44(1.21-4.90) | 0.012 | 157 | 23(14.6) | 134(85.4) | 1.78(0.78-4.07) | 0.173 |
| **AS in stock** |  |  |  |  |  |  |  |  |  |  |
| No | 91 | 24(26.4) | 67(73.6) | 1.0(ref) |  | 73 | 25(34.2) | 48(65.8) | 1.0(ref) |  |
| Yes | 276 | 54(19.6) | 220(80.4) | 1.74(0.80-3.81) | 0.164 | 257 | 33(12.8) | 224(87.2) | 5.35(1.61-17.82) | 0.006 |
| **Survey** |  |  |  |  |  |  |  |  |  |  |
| Baseline | 185 | 45(24.3) | 140(75.7) | 1.0(ref) |  | 164 | 32(19.5) | 132((80.5) | 1.0(ref) |  |
| Follow up | 182 | 33(18.1) | 149(81.9) | 1.57(0.89-2.74) | 0.118 | 166 | 26(15.7) | 140(84.3) | 1.46(0.75-2.86) | 0.259 |
